# Sodium Content of Lunches and Snacks Provided in Australian Long Day Care Centres: A Cross-Sectional Study

**DOI:** 10.3390/nu10030284

**Published:** 2018-02-28

**Authors:** Siobhan A. O’Halloran, Kathleen E. Lacy, Carley A. Grimes, Karen J. Campbell, Caryl A. Nowson

**Affiliations:** Institute for Physical Activity and Nutrition (IPAN), School of Exercise and Nutrition Sciences, Deakin University, 221 Burwood Highway, Burwood, VIC 3125, Australia; katie.lacy@deakin.edu.au (K.E.L.); carley.grimes@deakin.edu.au (C.A.G.); karen.campbell@deakin.edu.au (K.J.C.); caryl.nowson@deakin.edu.au (C.A.N.)

**Keywords:** children, childcare, sodium, menus, diet, salt

## Abstract

We determined the average amount of sodium provided in lunches and snacks and the average amount of sodium consumed at lunch in a convenience sample of Australian preschool children attending Long Day Care (LDC). Sodium content of lunches and snacks was determined from standardised recipes. Individual children’s sodium intake was estimated by a validated visual plate waste scale method. Five recipes (lunch *n* = 35, snacks *n* = 70) collected from 7 LDC centres; 95 children (50 boys) mean age 3.5 (SD) (0.2) years lunch intakes were assessed. Average total amount of sodium provided from two snacks and one lunch: 590 (146) mg, representing ~59% of the Australian Upper Level (UL) of intake (1000 mg/day sodium). Average total amount of sodium consumed: 541 (98) mg representing ~54% of the UL. Across all centres, the average sodium and energy consumed from lunch: 186 (108) mg (~19% of UL); 948 (437) kJ (38% of energy allowance); morning snacks: 63 (45) mg (6% of UL), 535 (183) kJ (21% of energy allowance); afternoon snacks: 291 (97) mg (29% of UL), 464 (171) kJ energy (46% of energy allowance). Australian LDC centres providing lunches cooked on site resulted in relatively low-sodium lunches.

## 1. Introduction

High dietary sodium intake is associated with high blood pressure (BP) [1]. Elevated BP follows a tracking pattern across the lifespan [2,3,4] and children who have raised BP are at increased risk of developing high BP and cardiovascular disease (CVD) as adults [5]. Reported average sodium intakes amongst children aged 1–5 years from economically developed countries such as the United Kingdom and United States are commonly high (1656 mg/day and 2070 mg/day, respectively), and most children’s intakes exceed dietary recommendations [6]. Dietary sodium intakes in Australian children aged 2–3 years have been estimated to be approximately 1500 mg/day (3.8 g salt/day) [7,8], which exceeds the daily Upper Level (UL) of Intake of 1000 mg/day by 50% for 1–3 years old [9]. In addition to raised BP, exposure to highly salted foods early in early life may also contribute to increased salt preference [10] and in turn may result in a habitually high salt diet persisting across life [11,12]. It is therefore important to begin lower salt eating patterns in early childhood.

In westernised countries the rising trend for both parents to be in the workforce, has resulted in increasing utilisation of out of home care [13]. Approximately half of Australian preschool children attend Long Day Care (LDC) centres [13], where they may typically consume half of their daily nutrients [14]. As such these setting play a potentially important role in children’s nutritional health. In recognition, a National Quality Framework sets Australian quality standards for early childhood and care and stipulates that food and drinks provided by early childhood centres are nutritious and appropriate for each child [15]. Within this standard there are no specific quantified recommendations for the amounts or types of foods that should be provided. However, within the State of Victoria, the Healthy Together Eating Advisory Service (HEAS), a State Government service, together with Nutrition Australia (Victorian State branch, Melbourne, Australia) has developed ‘The Menu Planning Guidelines’ for Victorian LDC centres [16]. These HEAS menu guidelines are based on the National Health and Medical Research Council’s (NH&MRC, Canberra, Australia) Australian Dietary Guidelines (ADG) [17] and recommend that the provision of lunch and morning and afternoon snacks to children aged 1–5 years should provide 50% of total daily energy and nutrient requirements [9,16,17]. Nutrition Australia/HEAS also offer a LDC menu review service where experienced nutritionists and dietitians evaluate LDC centre menus and recipes to ensure they meet the ADG. Nutrition Australia also offers an interactive workshop, which provides information on developing menus that meet ADG and nutrition requirements for preschool children.

Although Victorian LDCs may choose to be guided by the HEAS menu guidelines [16], there is no statutory requirement to adhere to the guidelines. Given the potential health impacts of sodium in children’s diets and the fact that many children spend substantial periods in care [13], it is important to assess the sodium content of the foods provided at LDC and the amount consumed by children. A few studies from the United States have reported that foods served in child care settings are high in sodium [18,19]. However, to date there is no information regarding the amount of sodium provided at Australian LDC centres. Therefore, this study aimed to determine the following: (i) the average amount of sodium provided in lunch and snacks; and (ii) the average amount of sodium consumed at lunchtime in a sample of Australian pre-school children attending LDC.

## 2. Methods

### 2.1. Study Design and Participants

The study sought to recruit LDC centres at which the food children were offered and consumed was measured. Informed by other dietary studies [20,21], this number was considered sufficiently large to provide dietary data representative of children attending LDC. Centres were eligible to participate if a cook worked on site, meals were provided to all children in care, and a conventional food system (raw food purchased and transformed into the final product for service) was used. To achieve this sample, LDC centre managers in the City of Greater Geelong (a regional city in the state of Victoria of ~200,000 inhabitants) were purposively selected (those centres within a distance of 35 km from the University). When centre managers declined to participate, another centre within the region was approached. This continued until the target number of centres (seven) was reached. Thirteen LDC centres (of a possible 42) were approached and seven agreed to participate (response rate 54%) Six centres (5 privately operated, 1 local government operated) declined to participate. The reasons cited reflected time scarcity (*n* = 2) and disinterest (*n* = 4). Ethical approval was obtained from the Deakin University Human Research Ethics Committee (ID number: HEAG-H 90/2015).

An initial phone call to centre managers gathered information on the number of 3–4-year-old children attending the centre, together with information on the facilities for food preparation and cooking practices. Managers of centres who prepared food on site, and who expressed interest participated in a face-to-face meeting with the researcher at the centre. At this meeting, managers provided information on their use of menu planning guidelines, whether menu reviews had been conducted, and whether staff had attended the Nutrition Australia menu planning workshop. Written consent from all LDC centre managers was also provided.

One week of recipes was collected from each of the centre cooks on the day of data collection (recipes were collected from the same week the observational data was collected) in 2016. Although centre lunch recipes included suggested servings per child (e.g., chicken fried rice recipe to serve 18 children) these servings differed from what was actually provided; thus, we have included data on the recipe suggested serving size as well as the observed portion provided to children, as described below.

### 2.2. Observational Data Collection

Observational data was collected from all children aged 3–4 years over one lunchtime period at each of the seven LDC centres in 2016.

Lunch meals were served using one of two systems:

1. Teacher-served lunch (5 centres)

A pre-portioned meal was served onto each child’s plate by the teacher and all children ate together. On request, second or third serves of lunch were portioned by the teacher.

2. Self-served lunch (2 centres)

Children decided when they were ready to eat over the lunchtime period and served themselves from the serving dish of lunch placed on the table. Second and third serves were permitted once all children at the table had finished their first serve and were portioned by the children.

Across all centres, dessert was provided after children had completed their lunch meal. When the dessert was fruit, children served themselves. When the dessert was yoghurt or custard, a pre-portioned serve was provided by the teacher.

### 2.3. Estimating the Portion of Lunch Provided on the Day of Data Collection

For centres where staff pre-plated lunch meals, the average weight of the first serve of the pre-portioned plated lunches was calculated from three randomly selected, sample portions (weighed to the nearest 1 g (calibrated scales UWE-2500) [22]. This estimated average weight of the initial serving of lunch dishes was summed with the actual weight of second and third serves, to give the total portion weight of lunch per child. For centres where children served themselves, the same calculation was applied: the average weights of the initial servings of self-served lunches were calculated from the average of three sample child-served portions and the actual weight of subsequent serves added to this to give total portion weight of lunch dishes. The average portion weight per child per centre was then calculated from the total portion weight of lunch per child (Table 1).

### 2.4. Photographing Lunch Portions

To identify each child’s portion size of lunch and dessert a unique identifying number was assigned to each child. Their plated lunch and dessert were digitally photographed prior to and following consumption to estimate the amount of lunch and dessert consumed. 

### 2.5. Estimating the Amount of Lunch Consumed

The amount of lunch consumed by each child was estimated from the average portion weight/child/centre lunch less the estimated amount left on the plate (determined from the photographed plate waste and using the validated plate waste method [22] (Table 2).

For example: 100 g (average portion weight/child/centre) –25% left on plate = 75 g lunch consumed.

### 2.6. Estimating the Amount of Sodium and Energy from Morning and Afternoon Snacks Provided

The amount of sodium and energy provided and consumed at lunchtime on the day of data collection were determined using the recipes provided by each centre, using the average portion size of the meals the children received or plated themselves and the amount consumed estimated from individual children’s plate waste. The average amounts of sodium and sodium density for each food item provided and consumed were assessed.

Dietary analysis of foods and ingredients used in recipes for the day of observation and for lunches, morning and afternoon snacks for one week (Monday-to-Friday) was performed using the dietary analysis program FoodWorks (Xyris Software; Brisbane, Australia). The average amount of sodium and energy, and energy density served and consumed at lunch was estimated on the day of observation expressed as an individual daily intake. Additionally, an estimate of the weekly average total amount of nutrients consumed for an individual child on one day was calculated using the average proportion of lunch consumed summed with the portions of snack served, assuming that 100% of snacks provided were consumed.

### 2.7. Comparison with Sodium Intake Recommendations

The amount of sodium provided and consumed per day per child from lunch and morning and afternoon snacks was compared with the National Health and Medical Research Council (NH&MRC, Canberra, Australia) Upper Level of Intake (UL) for sodium of 1000 mg/day (salt equivalent 2.5 g/day) for children aged 1–3 years (9) (e.g., lunch and 2 × snacks to provide 50% of UL for sodium = 500 mg sodium/day).

### 2.8. Estimate Energy Requirements (EER)

A Physical Activity Level (PAL) is used to estimate ones total energy expenditure [9]. The (PAL) of 1.55, corresponds to light activity and was used as the cut-off limit in the 2007 Australian National Children’s Nutrition and Physical Activity Survey [23]. In our study, a PAL of 1.55 was utilised to determine the recommended Estimated Energy Requirement (EER) of 5250 kJ/day for boys and 4900 kJ/day for girls aged 3 years [9]. The average EER of these two values (5000 kJ/day) was used to compare children’s estimated energy provided and consumed at LDC (i.e., lunch and 2 × snacks to provide 50% of total energy requirements = 2500 kJ energy/day) to recommendations.

### 2.9. Statistical Analysis

Descriptive statistics (mean (SD)) were used to describe sodium, sodium density and energy provided and consumed. Analysis of variance (ANOVA) was used to assess the differences in the average sodium and sodium density provided and consumed between lunch, morning, and afternoon snacks. Sidak post-hoc pairwise comparison was undertaken when ANOVA results indicated a significant difference across meal times. As sodium is correlated with energy, sodium density (sodium mg/MJ) was calculated to correct for differences in energy intake. Statistical analysis was performed using Stata14 software (Release; StataCorpLP, College Station, TX, USA). A *p*-value of <0.05 was considered significant.

## 3. Results

### 3.1. Children Attending Long Day Care

A total of seven LDC centres were recruited to participate in the study (council owned *n* = 5; privately owned *n* = 2). The teacher-served lunch system was used at five of the seven centres. Ninety-five preschool children aged 3–4 years were observed across the seven LDC centres over one lunchtime period. The average age of the participants was 3.5 years (SD 0.2) with a relatively even distribution of gender (males 53%).

### 3.2. Long Day Care Centre Menus

Nutrition Australia/HEAS reviewed recipes and menus from all seven centres. All centre managers and cooks voluntarily attended the Nutrition Australia workshop and utilised HEAS menu planning guidelines. Five days of recipes (lunch *n* = 35, snacks *n* = 70) were collected from centres (Supplementary Table S1). Over 5 days, six of seven centres provided pieces of fresh fruit for morning snack (i.e., apple, bananas and pears), while one centre provided muffins, pancakes, biscuits/rice cakes with ham and cheese for morning snack. This was offered at that centre three times during the week (or 3 of 5 days assessed) while fruit was provided on the other two days. Afternoon snacks across the centres were either cooked from raw ingredients (e.g., scones; cookies; carrot cake) or consisted of manufactured items (e.g., crackers and dips/cheese; raisin toast; bread and spreads); one centre provided fresh fruit for afternoon snack. All lunches comprised cooked mixed dishes, and dessert varied across the centres (e.g., fruit; yoghurt; apple pie; custard). Drinking water was the only drink provided across all centres.

### 3.3. Sodium, Sodium Density and Energy Provided from Lunch and Snacks

The average total amount of sodium, sodium density and energy provided to children from all lunches and snacks across all centres was 590 (SD) (146) mg, 1074 (135) mg/MJ and 2352 (453) kJ respectively. Across all centres, afternoon snacks had the highest sodium 291 (97) mg, which was almost twice sodium density 464 (171) mg/MJ (*p* = 0.001) from lunch: 194 (55) mg/MJ whereas the sodium density of the morning snack was lower 116 (46) mg/MJ (Table 3).

### 3.4. Sodium, Sodium Density and Energy Consumed from Lunch and Snacks

Children on average consumed 96% of energy and sodium provided. The average total sodium, sodium density and energy consumed by children from all lunches and snacks across all centres was 541 (SD 98) mg, 774 (129) mg/MJ and 2184 (236) kJ respectively (Table 4).

The amount of energy consumed at lunch is 37% of energy allowance for the age group compared to 28% provided by afternoon snacks. When assessing the average amount of sodium consumed at lunch, this was ~24% of the UL for sodium compared to afternoon snacks, which provided 29%.

## 4. Discussion

This study aimed to determine the average amount of sodium provided in lunch and snacks, and the sodium consumed from lunch in sample of Australian pre-school children attending LDC. In this sample of seven Australian LDC centres, the average sodium provided across all lunches and snacks was slightly higher (59%) and the total energy slightly lower (48%) than recommendations [16]. Children consumed on average 96% of lunch provided. Hence, the average total amount of sodium and energy consumed across one day of data collection was on average, less than that provided. This represented ~54% of the UL of intake of 1000 mg/d for sodium for 1–3 years old and 87% of the recommended 5000 kJ EER for children aged 3 years. The Healthy Together Eating Advisory Service (HEAS) recommends that the 2 snacks and lunch served at LDC should provide approximately 50% of daily nutrient requirements [16]. As sodium is the only nutrient for which excess intakes are the concern, rather than inadequate intakes, an achievable target would be to ensure that the sodium content of the foods provided daily at LDC centres does not exceed half of the UL for sodium, i.e., 500 mg of sodium [9]. Given that national survey data has shown that Australian preschool children’s sodium intakes are high and exceed the UL by one and half times, the sodium content of the food provided at LDC centres, although slightly higher than the UL, was not excessive. It was clear that cooking from fresh low-sodium ingredients resulted in a lunch with a relatively low sodium content, which is in contrast to the afternoon snack, which included a number of processed foods, raising the sodium intake to a higher level.

Consistent with the Australian Dietary Guidelines (ADG) [17], the HEAS menu planning guidelines stipulate that the addition of salt during cooking or at the table should be avoided in LDCs and that low/reduced salt or no added salt packaged foods (e.g., sauces, stock or canned products) should be used during cooking [16]. The menu guidelines further note that ham and bacon, (which contribute substantially to Australian children’s dietary sodium intakes [7,8]), should be limited to less than twice a week and that sausages, frankfurts and salami, and discretionary food items (e.g., chips and savoury snacks), should also be avoided [16,17]. Importantly, all centres in our study had voluntarily applied the HEAS guidelines [16] recommendation to use low/reduced-salt or no-added-salt packaged products, such as low-sodium canned varieties of tomatoes, fish and legumes, stock cubes, and had limited the use of cured meats to once or twice a week during the preparation of lunch and afternoon snacks. This is important, as according to a systematic review of the sodium content of processed foods on the Australian market, processed meats contain the second-highest mean sodium content (846 mg/100 g) (after sauces and spreads). In addition, the sodium content of canned products can vary ranging from 0 mg to 782 mg/100 g for canned vegetables and legumes and from 47 mg/100 g 1170 mg/100 g for canned fished [24]. Selecting lower-sodium alternatives for use at LDC can therefore likely make a difference to the amount of sodium preschool children consume over the course of the day.

The average amount of sodium provided per child from lunches was 236 (139) mg, representing ~24% of the UL of intake for sodium. Although there is no guidance as to how the UL of 1000 mg/day for sodium should be apportioned over three meals and snacks, as a proportion of the UL, it is reasonable to assume one quarter of the recommended 1000 mg/day (i.e., ~250 mg) for each of breakfast, lunch, dinner and collectively three snacks. Hence, this average sodium from the provided lunches is slightly lower than 25% of the UL for sodium. These levels of sodium were achieved because lunches were made from raw ingredients (e.g., lasagne; chicken curry; ten vegetable pasta bake) which included a variety of fresh or frozen vegetables (e.g., tomatoes, carrots, pumpkin). These ingredients are naturally low in sodium [25] and therefore resulted in lunch dishes with lower average sodium contents (lowest was 66 (68) mg). Only in two of the seven centres did the sodium content (~400 mg/day) of the lunch consumed exceeded 250 mg (quarter of the UL), which was driven by the use of high sodium ingredients such as taco seasoning and Worcestershire sauce™.

It is also important to note the frequent use of fresh foods in recipes utilised in LDC lunches are different to the foods children receive once they start primary school. Australian school children frequently either bring home prepared sandwiches or purchase them from the school canteen [26]. Sandwiches often consist of bread, cheese and ham [27], which are key sources of sodium in school-aged children, together contributing ~26% of total daily sodium [7,8]. In addition, findings from a cross-sectional study of national survey data revealed that amongst children aged 6–16 years, lunch was the most sodium dense meal of the day (363 mg/MJ), compared to dinner and breakfast [28]. The transition from preschool to primary school therefore represents an opportunity to limit the shift from low-to-high-sodium dense foods, which likely could be achieved by strategies such as a comprehensive reformulation of lower-sodium foods [29] and providing parents with ideas for lower-salt lunch options.

Of the two snacks and lunches provided, afternoon snacks provided the highest average amount of sodium (291 (97) mg) (29% of the UL) and sodium density (464 (171) mg/MJ) across the seven centres. Snacks at the majority of centres in this study comprised relatively highly salted foods including cheese and ham, rice crackers, tomato and cheese pizzas, toast/English muffins/crumpets with cheese and Vegemite (“Kraft”) (highly salted yeast spread) (one centre, on one occasion, provided fruit for afternoon snack). One centre provided a range of processed foods such as ham and cheese rice crackers, muffins and pancakes for morning snack on five occasions and consequently morning snacks at this centre provided an average amount of sodium (166 (45) mg) that was approximately three times higher than morning snacks provided at the other six centres. This indicates that it is challenging to provide a range of lower-salt foods for mid-meal snacks even for these centres, which applied the HEAS menu planning guidelines and provides evidence affirming the need to reduce the amount of sodium in the food supply. To assist further with the provision of afternoon snacks, HEAS guidelines could also be reviewed to include more examples of low sodium foods available and how to choose these options. Furthermore, a number of ingredients used in afternoon snacks were breads, cheese, ham and crackers, which, according to the 2011-13 AHS, are major sources of dietary sodium in children aged 2–3 years, contributing up to 16% of total daily sodium intake [7]. These ingredients can taste salty, and may have an effect on increasing children’s taste preference for salty foods, leading to a lifetime avidity for salt [11,12]. Hence afternoon snack time represents an opportunity to reduce preschool children’s exposure of high salt foods by providing them with more fresh foods (e.g., vegetable sticks served with homemade dips made at the centres) and less cheese and crackers.

In our sample, we found that morning snacks contributed the least amount of sodium (63 (45) mg) (6% of the UL) and were the least sodium dense (116 (46) mg/MJ) as most of these foods included a variety fresh fruits and cow’s milk. According to the 2011-13 Australian Health Survey fresh fruit and dairy milk contributed only moderate amounts to total dietary sodium intakes in children aged 2–3 years (~7% and 0.3% respectively) (7). Moreover, these foods were the main contributors to dietary potassium in young Australian children’s diets, and provided together ~40% of total dietary potassium. Throughout childhood, potassium is a key moderator of blood pressure, and longitudinal studies have shown that a higher intake of potassium is associated with lower systolic blood pressure [30,31]. Furthermore, the ADG recommends one serve of fruit and one serve of dairy for children aged 2–3 years [17] as important sources of dietary potassium. Our findings were in good agreement with a previous Australian cross-sectional study and our own subsequent study (utilising the same dataset), which found that the majority of surveyed LDC centres provided the recommended servings of fruit and dairy (i.e., one serve/day) [32,33]. This was, however, in contrast to data from the United States, which showed that children were served inadequate amounts fruit [21,22] and foods served at snack time were high in fat [19,22] and added sugars [34]. It is therefore reassuring that most morning snacks provided in this sample of Australian LDC centres with the facilities and staff to prepare foods were based principally on fresh fruits and milk.

The analysis of five days of recipes from each centre allowed for the assessment of typical foods, beverages and ingredients provided to children at these settings on a regular basis. However, we did not measure the actual sodium content of food served, and relied on recipes and reports from the cooks relating use of salt; therefore, it would be useful to undertake some chemical analysis of the salt content of these meals to confirm these findings. It is acknowledged that there are variations in the sodium content across different brands of foods, and not all branded food items were included in the food database. Similar but not exact branded products were used in the analysis. We also used just one day of observational dietary data to assess the amounts of sodium and food groups actually consumed andalthough it seems unlikely, the amount of food consumed by the children on the day of observation could have been different from the other days, although we did capture large inter- individual variations in intake. Our findings cannot be generalised to other Australian LDC centres as all seven centres were within the same locality and all adhered to the HEAS menu planning guidelines. It is possible that LDCs who consented to participation in this study were more interested in providing foods that met national standards than those that did not. As such, these data are likely to present the best-case scenario regarding the contribution to total sodium intake. It is likely that centres that do not participate in programs that promote healthy eating will have substantially higher sodium content menus. This highlights the importance of broader assessment of the foods provided and eaten in LDC and including in future centres, which do not have onsite cooking facilities and/or staff trained in food preparation and those which do not follow the HEAS menu guidelines.

## 5. Conclusions

Our study found that the average total amount of sodium and energy provided and consumed from morning snacks, lunch and afternoon snacks at Australian LDC centres that prepared their own lunches resulted in relatively low-sodium lunches, which were acceptable to children and enabled their dietary sodium intake to remain close to half of recommended upper limit for sodium whilst in care. However, foods provided particularly as an afternoon snack contained a relatively high amount of sodium due to use of manufactured foods such as cheese, biscuits, bread, ham and Vegemite.

## Figures and Tables

**Table 1:** An example of the derivation of estimated total portion weight of lunch per child and the average portion weight per child per centre.

Child	Average Serving Size Per Child (g)	Actual Weight of Second Serving (g)	Actual Weight of Third Serving (g)	Total Portion Weight of Lunch Per Child (g)
1	165	0	0	165
2	125	118	0	243
3	125	120	122	367
Average portion weight/child/centre (g)				258

**Table 2:** Visual plate waste seven-point scale representing percent waste used to estimate the amount of lunch consumed [22].

Plate Waste	Percentage
None left	0
One mouthful left	10
¼ left	25
½ left	50
¾ left	75
One mouthful eaten	90
All left	100

**Table 3:** The average (mean (SD)) Na (sodium mg) and energy (kJ) provided to children over a five-day menu cycle, from morning snack (MS), lunch and afternoon snack (AS) at seven long day care centres.

	Morning Snack			Lunch			Afternoon Snack					
	Na (mg)	Na density (mg/MJ)	Energy (kJ)	Na (mg)	Na Density (mg/MJ)	Energy (kJ)	Na (mg)	Na Density (mg/MJ)	Energy (kJ)	Total Sodium (mg)	Total Energy Density (mg/MJ)	Total Energy (kJ)
Centre 1 ^a^	40 (0.5)	94 (4)	428 (17)	135 (82)	194 (126)	719 (134)	386 (335)	660 (478)	612 (206)	561	943	1759
Centre 2	50 (0.9)	113 (7)	437(25)	220 (61)	140 (52)	1614 (208)	331 (199)	537 (302)	578 (118)	601	789	2629
Centre 3	40 (0.7)	97 (11)	427 (66)	419 (308)	297 (164)	1333 (249)	389 (188)	586 (182)	642 (222)	848	985	2402
Centre 4 ^a^	166 (55)	219 (29)	936 (330)	66 (68)	151 (112)	433 (145)	154 (147)	174 (123)	824 (394)	386	560	2193
Centre 5	48 (1)	89 (5)	557 (32)	164 (60)	152 (24)	1093 (396)	357 (219)	516 (180)	652 (238)	569	757	2302
Centre 6	49 (0.7)	100 (9)	495 (53)	220 (124)	203 (154)	1205 (410)	220 (109)	482 (356)	900 (593)	489	773	2600
Centre 7	51 (0.8)	105 (7)	468 (34)	429 (168)	220 (110)	1415 (650)	199 (43)	292 (77)	697 (111)	679	609	2580
Average across centres	63 (45)	116 (46)	535 (183)	236 (139)	194 (55)	1116 (543)	291 (97)	464 (171)	701(118)	590 (146)	774 (156)	2352(453)

^a^ Centre which used self serve lunch.

**Table 4:** The estimated average amount of Na (sodium mg) and energy (kJ) consumed at lunch on the observation day combined with amount of sodium provided from morning snack (MS), lunch and afternoon snack (AS) at seven long day care centres (mean (SD)).

	Morning Snack			Lunch			Afternoon Snack					
	Na (mg)	Na Density (mg/MJ)	Energy (kJ)	Na (mg)	Na Density (mg/MJ)	Energy (kJ)	Na (mg)	Na Density (mg/MJ)	Energy (kJ)	Total Sodium (mg)	Total Na Density (mg/MJ)	Total Energy (kJ)
Centre 1 ^a^	40 (0.5)	94 (4)	428 (17)	118	140	842	386 (335)	660 (478)	612 (206)	544	894	1882
Centre 2	50 (0.9)	113 (7)	437(25)	180	121	1489	331 (199)	537 (302)	578 (118)	561	771	2504
Centre 3	40 (0.7)	97 (11)	427 (66)	213	266	799	389 (188)	586 (182)	642 (222)	642	949	1868
Centre 4 ^a^	166 (55)	219 (29)	936 (330)	66	151	433	154 (147)	174 (123)	824 (394)	386	544	2193
Centre 5	48 (1)	89 (5)	557 (32)	162	160	1011	357 (219)	516 (180)	652 (238)	567	765	2220
Centre 6	49 (0.7)	100 (9)	495 (53)	167	196	850	220 (109)	482 (356)	900 (593)	436	778	2245
Centre 7	51 (0.8)	105 (7)	468 (34)	400	329	1215	199 (43)	292 (77)	697 (111)	650	726	2373
Average across the centres	63 (45)	116 (46)	535 (183)	186 (108)	194 (70)	948 (437)	291 (97)	464 (171)	701(118)	541 (98)	774 (129)	2184 (236)

^a^ Centres which used self serve lunch.

## Supplementary Materials

The following are available online at http://www.mdpi.com/2072-6643/10/3/284/s1, Table S1: One week menu for the seven Australian long day care centres.
